# The Impact of Aluminum Doping on the Performance of MgV_2_O_4_ Spinel Cathodes for High-Rate Zinc-Ion Energy Storage

**DOI:** 10.3390/molecules30132833

**Published:** 2025-07-01

**Authors:** He Lin, Zhiwen Wang, Yu Zhang

**Affiliations:** A State Key Laboratory of Chemistry and Utilization of Carbon Based Energy Resources, School of Chemistry, Xinjiang University, Urumqi 830017, China

**Keywords:** zinc-ion batteries, cathode materials, vanadium oxide, aluminum doping

## Abstract

This study explores the development of aluminum-doped MgV_2_O_4_ spinel cathodes for aqueous zinc-ion batteries (AZIBs), addressing the challenges of poor Zn^2+^ ion diffusion and structural instability. Al^3+^ ions were pre-inserted into the spinel structure using a sol-gel method, which enhanced the material’s structural stability and electrical conductivity. The doping of Al^3+^ mitigates the electrostatic interactions between Zn^2+^ ions and the cathode, thereby improving ion diffusion and facilitating efficient charge/discharge processes. While pseudocapacitive behavior plays a dominant role in fast charge storage, the diffusion of Zn^2+^ within the bulk material remains crucial for long-term performance and stability. Our findings demonstrate that Al-MgV_2_O_4_ exhibits enhanced Zn^2+^ diffusion kinetics and robust structural integrity under high-rate cycling conditions, contributing to its high electrochemical performance. The Al-MgVO cathode retains a capacity of 254.3 mAh g^−1^ at a high current density of 10 A g^−1^ after 1000 cycles (93.6% retention), and 186.8 mAh g^−1^ at 20 A g^−1^ after 2000 cycles (90.2% retention). These improvements, driven by enhanced bulk diffusion and the stabilization of the crystal framework through Al^3+^ doping, make it a promising candidate for high-rate energy storage applications.

## 1. Introduction

The escalating global demand for energy, coupled with growing environmental pollution, poses a significant challenge, exacerbating both the energy crisis and environmental degradation [1]. In response, the pursuit of carbon neutrality has emerged as a key priority in global agendas. The increasing concentration of carbon dioxide is a major catalyst for the rapid expansion of renewable energy sources, such as solar and wind power [2,3]. However, the efficient integration of these renewable sources into the energy grid is contingent upon their effective transportation and storage [4]. In this context, electrochemical energy storage systems, particularly batteries, are regarded as critical for the storage of renewable energy [5]. Lithium-ion batteries (LIBs), which have long been the dominant technology for energy storage, are widely utilized in portable electronics, including smartphones and laptops, as well as in large-scale applications such as electric vehicles [6]. The high energy density and extended cycle life of LIBs have contributed to their attractiveness. However, despite these advantages, the long-term sustainability of LIBs is constrained by several challenges, including the finite global reserves of lithium, safety concerns, and high production costs [7,8,9]. These limitations raise concerns about the viability of LIBs for widespread adoption in the coming decades.

In light of the limitations of LIBs, there is an increasing focus on alternative battery technologies that utilize more abundant and environmentally benign materials, such as sodium (Na^+^) [10], potassium (K^+^) [11], and multivalent ions, including magnesium (Mg^2+^) [12], zinc (Zn^2+^) [13]. Among these, aqueous zinc-ion batteries (AZIBs) have attracted significant attention due to their promising electrochemical characteristics. Zinc is not only abundant and cost-effective but also amenable to direct use as an anode material, which substantially reduces manufacturing costs. Additionally, zinc exhibits a relatively high theoretical capacity of 820 mAh g^−1^ and a redox potential of −0.763 V versus the standard hydrogen electrode (SHE), making it a compelling alternative for energy storage [14,15,16,17,18,19,20,21]. AZIBs utilize slightly acidic or near-neutral aqueous electrolytes (pH 3.6–6.0), which enhance cycle safety and contribute to improved reversible capacity. The high ionic conductivity of aqueous electrolytes (1 S cm^−1^) in comparison to organic electrolytes (1 to 10 mS cm^−1^) facilitates faster ion migration, potentially enhancing the overall charging and discharging performance. As a result, AZIBs offer a promising, environmentally friendly, and sustainable alternative to LIBs for large-scale energy storage. However, despite their advantages, the development of suitable cathode materials for AZIBs remains a significant challenge. Issues such as cathode dissolution, structural instability during charge and discharge cycles, inefficient zinc ion intercalation and deintercalation, and slow reaction kinetics persist, highlighting the urgent need for the development of advanced cathode materials that demonstrate high specific capacity, improved rate performance, and enhanced cycle stability [22,23,24,25].

Recent research on cathode materials for AZIBs has primarily focused on manganese-based compounds [26,27,28], vanadium-based compounds [29,30,31,32], Prussian blue analogs (PBAs) [33,34,35], and organic materials [36,37]. Among these, vanadium-based materials, particularly vanadium oxides, have garnered significant interest due to their ability to exhibit multiple valence states (+5, +4, +3, +2) and a variety of crystal structures, including layered, quasi-layered, and tunnel configurations [38,39,40,41]. These structural variations present opportunities for the design of novel electrode materials with distinct and tunable electrochemical properties [42,43]. Despite their promising characteristics, vanadium-based cathode materials are hindered by several challenges, including poor electrical conductivity, low ion diffusion coefficients, and structural instability during charge and discharge cycles.

One promising approach to overcoming these challenges is the use of spinel-type vanadium oxide structures. Spinel materials, known for their extensive internal three-dimensional space, are capable of accommodating ion insertion and have been widely utilized in various battery systems, including LIBs [44,45,46]. In the context of AZIBs, spinel materials such as ZnMn_2_O_4_ [47], ZnCo_2_O_4_ [48], and MgMn_2_O_4_ [49] have been explored; however, vanadium-based spinel cathode materials have been less extensively investigated.

Spinel-type MgV_2_O_4_ (MgVO), a vanadium-based ternary oxide, has garnered significant attention as a promising cathode material for AZIBs due to its robust framework, redox-active vanadium centers, and structural tunability. Recent studies have revealed that MgVO exhibits a relatively high degree of structural stability under electrochemical cycling, attributed to the rigid spinel lattice that accommodates the reversible intercalation and deintercalation of Zn^2+^ ions. For instance, electrochemical activation of MgVO during prolonged cycling has been reported to enhance the oxidation state of vanadium, thereby improving the electrochemical activity and contributing to a notable reversible capacity of 128.9 mAh g^−1^ after 500 cycles at a high current density of 4.0 A g^−1^ [50].

Despite these favorable features, a key limitation lies in the sluggish Zn^2+^ diffusion within the spinel matrix, which is largely governed by the strong electrostatic interactions between the divalent Zn^2+^ ions and the negatively charged oxygen framework. These interactions hinder the ion transport kinetics and reduce the overall rate capability of the material [51,52,53].

To overcome these challenges, various doping strategies have been explored. Notably, the incorporation of metal cations such as Zn^2+^ and Mg^2+^ into the vanadium oxide lattice has shown potential in modulating the local electronic environment and mitigating the electrostatic repulsion between Zn^2+^ and the host structure [52,53]. Such dopants can partially occupy tetrahedral or octahedral sites within the spinel framework, leading to lattice distortion, expanded interstitial spaces, and enhanced electronic conductivity. These structural modifications collectively improve Zn^2+^ diffusion kinetics and cycle stability.

Aluminum (Al) is one of the most abundant metals in the earth’s crust and offers several advantages over transition metals, including low cost and low toxicity. The Al^3+^ ion forms strong Al–O bonds with oxygen atoms, with bond energies significantly higher than those of Na–O, K–O, and other alkaline metal–O bonds. These properties make aluminum an attractive candidate for improving the performance of vanadium-based cathodes [54].

Motivated by these considerations, we employed a sol-gel method to pre-insert Al^3+^ ions into the spinel MgVO structure, thereby enhancing its structural stability and improving its conductivity. The Al^3+^ ions form Al–O bonds, which mitigate the electrostatic interactions between Zn^2+^ ions and the host material, facilitating the intercalation and deintercalation of Zn^2+^ ions. The resulting Al-doped MgVO cathode material (Al-MgVO) exhibits improved ion diffusion, enhanced conductivity, and excellent electrochemical performance. At an ultra-high current density of 10 A g^−1^, the material retains a capacity of 254.3 mAh g^−1^ after 1000 cycles, with a capacity retention of 93.6%. Additionally, at 20 A g^−1^, the material maintains a capacity of 186.8 mAh g^−1^ after 2000 cycles, with a capacity retention of 90.2%, demonstrating its potential for high-rate applications.

## 2. Results and Discussion

### 2.1. Morphological Characterization

The Al-MgVO composite and MgVO were synthesized using the sol-gel method. The elemental composition of these materials was determined through Inductively Coupled Plasma (ICP) analysis, which provided precise quantification of the major elements present in the samples. The results, summarized in Table 1, offer valuable insights into the composition of the synthesized materials.

For the Al-MgVO composite, the ICP analysis yielded the chemical formula Al_0.26_Mg_1.23_V_2_O_4_, confirming the successful incorporation of Al^3+^ ions into the MgVO framework, as anticipated. The substitution of Mg^2+^ with Al^3+^ is clearly reflected in the increased concentration of aluminum in the composite material. The molar ratio of Al to Mg (0.26:1.23) aligns with the intended doping strategy, where Al^3+^ ions partially replace Mg^2+^ sites within the MgVO structure. This doping process is expected to enhance the electrochemical properties of the material, particularly by improving ion diffusion and structural stability. In contrast, the ICP analysis of the pure MgVO sample, presented in Table 1, revealed the chemical formula MgV_2_O_4_, which is consistent with the expected stoichiometry for undoped MgVO, confirming the absence of unintended elements.

A significant observation from the ICP results is that the doping of Al^3+^ leads to an increase in the Mg content, accompanied by a corresponding decrease in the vanadium (V) content. This shift suggests that the incorporation of Al^3+^ into the lattice may alter the local environment of the V atoms, potentially modifying the charge distribution within the structure. These findings indicate that Al doping could influence the stability of the vanadium oxide framework, thereby enhancing its electrochemical performance by mitigating excessive vanadium dissolution during cycling.

**Figure 1:**
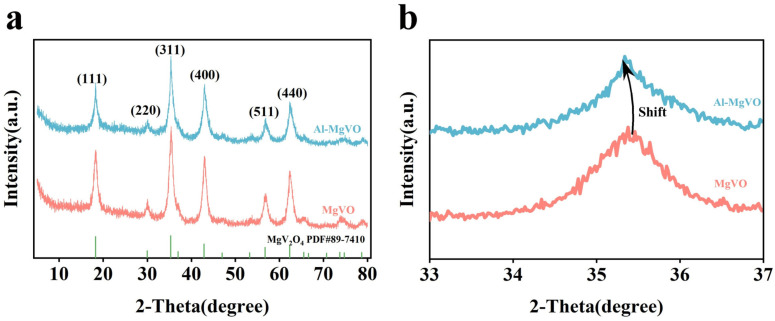
(**a**) XRD diffraction of MgVO and Al-MgVO; (**b**) enlarged view of the diffraction peak (311).

In-depth comparison of the XRD patterns for pristine MgVO and Al-doped MgVO (Al-MgVO) reveals important insights into the structural effects induced by Al doping. As shown in Figure 1a, both materials exhibit diffraction peaks that are well indexed to the spinel-type MgV_2_O_4_ phase (JCPDS No. 89-7410), confirming that the main crystal structure remains unaltered upon Al incorporation. The absence of impurity peaks or secondary phases further demonstrates the high phase purity and successful synthesis of both samples.

Closer examination of Figure 1b uncovers a discernible shift in the (311) diffraction peak, which moves from 35.6° in pristine MgVO to 35.3° in Al-MgVO. This peak shift can be attributed to the substitutional doping of Mg^2+^ ions by smaller Al^3+^ ions within the crystal lattice. Although the ionic radius of Al^3+^ (0.54 Å) is smaller than that of Mg^2+^ (0.72 Å), the observed peak shift toward lower 2θ values indicates a slight lattice expansion. This counterintuitive result may be due to local structural distortions or changes in the electronic environment surrounding the vanadium ions upon Al incorporation.

The increased interplanar spacing of the (311) planes, as inferred from the peak shift, suggests that Al^3+^ doping introduces subtle but meaningful modifications to the crystal lattice. Such changes can influence the diffusion pathways for Zn^2+^ ions, potentially enhancing electrochemical performance by facilitating ion transport and improving structural stability during cycling. These XRD findings provide clear evidence that Al doping is achieved without altering the fundamental spinel framework, while simultaneously introducing beneficial lattice modifications that may contribute to the superior properties of Al-MgVO as a cathode material.

**Figure 2:**
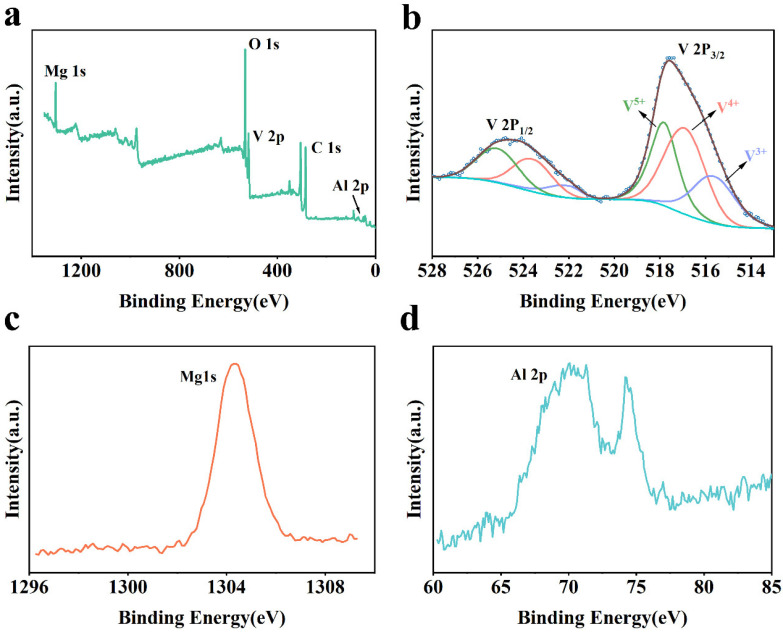
(**a**) XPS full spectrum of Al-MgVO; (**b**) XPS high-resolution spectrum of V 2p; (**c**) XPS spectrum of Mg 1s; (**d**) XPS spectrum of Al 2p.

**Figure 3:**
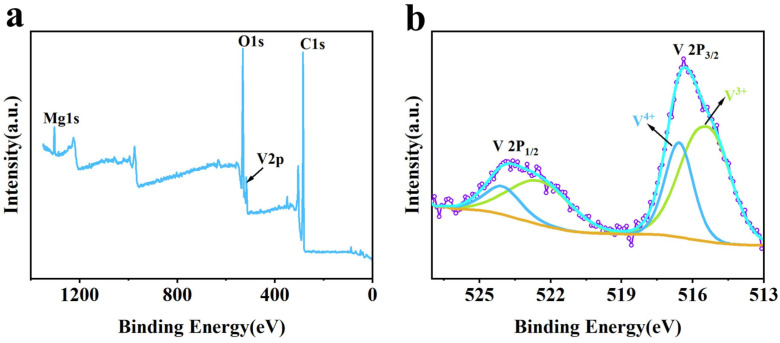
(**a**) XPS full spectrum of MgVO; (**b**) XPS high-resolution spectrum of V 2p.

To further elucidate the chemical environment and valence states of the constituent elements, a comparative XPS analysis was performed on both Al-doped MgVO (Al-MgVO) and undoped MgVO samples. The full XPS spectra (Figure 2a and Figure 3a) confirm the elemental composition of each sample: Al, Mg, V, and O are detected in Al-MgVO, while only Mg, V, and O are observed in MgVO, validating the absence of Al in the pristine material.

The high-resolution V 2p XPS spectra of Al-MgVO and undoped MgVO provide valuable insights into the vanadium oxidation states, offering a clear distinction between the two materials and the effects of Al^3+^ doping on vanadium’s electronic structure.

For Al-MgVO (Figure 2b), the V 2p spectrum displays six distinct peaks, which are associated with V^3+^, V^4+^, and V^5+^ oxidation states. These peaks are observed at 516.5 eV, 522.1 eV (V^3+^), 516.9 eV, 523.6 eV (V^4+^), and 517.8 eV, 525.1 eV (V^5+^). The most prominent peak at 516.9 eV corresponds to the V 2p_3/2_ signal for V^4+^, indicating that V^4+^ is the dominant oxidation state in Al-MgVO. This suggests that the introduction of Al^3+^ into the MgVO lattice promotes the stabilization of V^4+^ within the spinel structure. The presence of V^3+^, as evidenced by the peaks at 516.5 eV and 522.1 eV, confirms the coexistence of multiple oxidation states, indicating a mixed-valence environment. This mixed-valence state is important because it is known to enhance electron conductivity, as the presence of multiple oxidation states facilitates electron hopping. Furthermore, the co-existence of V^3+^ and V^4+^ contributes to increased redox activity, which may be beneficial for the electrochemical performance of the material, particularly during charge and discharge cycles in zinc-ion batteries.

In stark contrast, the V 2p spectrum of undoped MgVO (Figure 3b) shows two major sets of peaks at 515.3 eV/522.4 eV and 516.6 eV/524.1 eV, which correspond to V^3+^ and V^4+^, respectively. The higher intensity of the V^3+^-related peaks suggests that the pristine MgVO primarily contains V^3+^ species, in alignment with the expected stoichiometry of the spinel MgV_2_O_4_ structure, where vanadium predominantly exists in the +3 oxidation state. This observation is in agreement with previous studies on MgVO, where V^3+^ is known to be the dominant valence state.

The key distinction between Al-MgVO and MgVO lies in the relative amounts of V^4+^. The reduced presence of V^4+^ in undoped MgVO underscores the significant role of Al^3+^ doping in modulating the vanadium valence state. The higher proportion of V^4+^ in Al-MgVO is indicative of the ability of Al to influence the electronic structure and increase the overall charge carrier density within the material. This is especially crucial for enhancing the material’s conductivity and facilitating efficient electron transfer during the electrochemical processes.

The high-resolution Mg 1s spectrum of the Al-MgVO sample (Figure 2c) presents a single peak centered at 1304.18 eV, which is consistent with the binding energy characteristic of Mg^2+^ species in a spinel lattice. This observation suggests that Al incorporation does not significantly alter the chemical environment of Mg in the lattice, as no shift indicative of a change in the oxidation state of Mg is observed. The lack of a noticeable chemical shift further confirms that the Mg ions retain their +2 oxidation state, and Al incorporation occurs without causing substantial distortions in the local Mg environment.

The Al 2p spectrum (Figure 2d) of the Al-MgVO sample reveals two distinct peaks located at binding energies of 71.2 eV and 74.2 eV. The peak at 71.2 eV is assigned to tetrahedrally coordinated Al^3+^ species, which is a typical feature for aluminum in spinel structures [55]. The peak at 74.2 eV corresponds to Al–O bonding environments, indicating the presence of aluminum species that are chemically bonded with oxygen atoms within the structure [56,57]. This finding supports the hypothesis that Al does not only substitute for Mg in the lattice but also interacts with the oxygen ions, potentially forming Al–O bonds, which might contribute to enhanced structural integrity and stability of the material.

The presence of both tetrahedral Al^3+^ species and Al–O bonding environments suggests that Al plays a dual role within the Al-MgVO structure. On one hand, Al substitution for Mg^2+^ in the spinel lattice helps maintain the overall crystal structure and may influence electrochemical properties. On the other hand, the formation of Al–O bonds may provide additional stabilization to the material, potentially improving the cycling performance and enhancing the structural integrity of the Al-MgVO cathode material. This dual effect of Al doping could be advantageous in optimizing the electrochemical behavior of Al-MgVO for use in zinc-ion batteries, providing both structural and electronic benefits.

**Figure 4:**
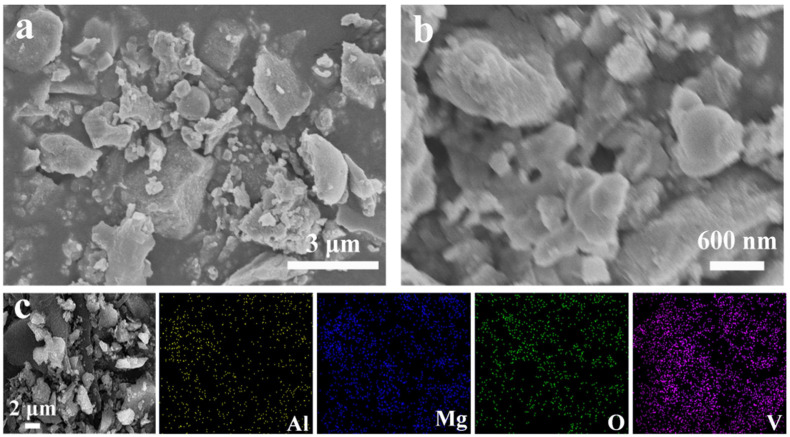
(**a**,**b**) SEM patterns of Al-MgVO; (**c**) EDS mapping of Al-MgVO.

The surface morphology of the Al-MgVO sample was characterized using scanning electron microscopy (SEM), and the results are presented in Figure 4. Figure 4a,b show the SEM images of Al-MgVO at different magnifications. The Al-MgVO sample exhibits a dispersed surface morphology composed of irregular block-like structures and microspheres with inconsistent sizes ranging from 1 to 3 µm. Upon further magnification in Figure 4b, nanoscale microspherical structures can be observed on the surface of the block-like particles. This nanoscale feature is likely a result of aggregation during the synthesis process, leading to the formation of both dispersed spherical and irregular block-like structures at the micron scale. The presence of nanoscale microspheres on the block surface is beneficial for increasing the contact area between the cathode material and the electrolyte, which can enhance the electrical conductivity and overall electrochemical performance of the material. Additionally, Energy-Dispersive Spectroscopy (EDS) mapping shown in Figure 4c clearly illustrates the homogeneous distribution of Al, Mg, V, and O elements within the Al-MgVO structure, further confirming the successful incorporation of aluminum into the matrix and its uniform distribution throughout the material.

### 2.2. Electrochemical Properties Characterization

The zinc-ion storage performance of the Al-MgVO material was carefully evaluated through galvanostatic charge–discharge (GCD) and cyclic voltammetry (CV) tests, which provided insights into the material’s electrochemical characteristics and activation behavior.

**Figure 5:**
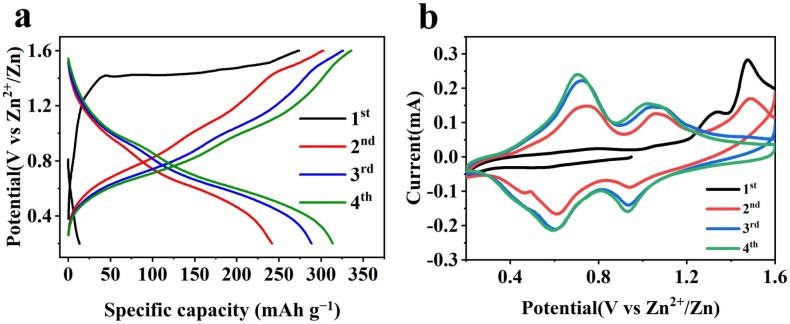
(**a**) Charge and discharge curve of the first four rounds of Al-MgVO; (**b**) the CV cycle curve corresponding to (**a**).

In the GCD analysis (Figure 5a), the first charge cycle reveals a stable plateau around 1.5 V, which is characteristic of the reversible redox process involving the insertion and extraction of zinc ions within the Al-MgVO structure. This initial plateau is particularly important, as it suggests the activation of the Al-MgVO material, where the structural framework undergoes a modification that facilitates zinc ion intercalation. This initial plateau disappears in subsequent cycles, reflecting the stabilization of the electrochemical processes after the activation step. Notably, the initial charge capacity of 273.9 mAh g^−1^ is much higher than the initial discharge capacity, indicating a significant amount of energy is stored during the first charge. This discrepancy is attributed to the material’s activation during the first cycle, wherein the formation of a stable electrochemical interface and potential structural reorganization occurs. Such an activation step is crucial for the establishment of a stable, reversible cycling behavior in later cycles.

As the cycling progresses, the charge and discharge curves gradually converge, which indicates excellent reversibility and minimal capacity fading over multiple cycles. This behavior is a hallmark of the material’s high stability and suggests that the Al-MgVO material reaches an equilibrium state after the initial activation, with minimal irreversible loss of capacity in subsequent cycles. This stability is indicative of the potential of Al-MgVO as a reliable cathode material for AZIBs, capable of maintaining long-term performance.

In the CV analysis (Figure 5b), the first cycle exhibits a prominent oxidation peak around 1.5 V, which correlates with the charge plateau observed in the GCD curve. This peak represents the electrochemical process of Zn^2+^ insertion into the Al-MgVO structure, further confirming the activation behavior seen in the GCD results. As the number of cycles increases, this oxidation peak diminishes significantly, disappearing entirely in the later cycles. This reduction and eventual disappearance of the oxidation peak serve as evidence of the stabilization of the electrochemical processes, consistent with the completion of the initial activation. The disappearance of the peak signifies that the material undergoes a conditioning phase during the first few cycles, where the electrochemical processes become more stable and efficient, supporting the idea of reversible Zn^2+^ intercalation and de-intercalation within the Al-MgVO framework.

**Figure 6:**
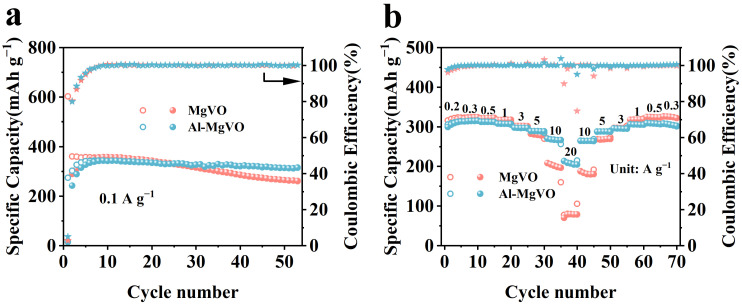
(**a**) Cycling performance of MgVO and Al-MgVO at 0.1 A g^−1^. (**b**) Rate performance of MgVO and Al-MgVO at different current densities. Open symbols (regardless of shape, such as circles or stars) represent the charge process, while solid symbols indicate the discharge process.

To comprehensively evaluate the electrochemical performance of the synthesized materials, the cycling behaviors of Al-MgVO and pristine MgVO cathodes were investigated at a current density of 0.1 A g^−1^, as illustrated in Figure 6a. The Al-MgVO electrode exhibited an unusually low initial discharge capacity of 13.5 mAh g^−1^, followed by a rapid increase to a charge capacity of 338.5 mAh g^−1^, and eventually reached 341.6 mAh g^−1^ after several cycles. After seven cycles, the Coulombic efficiency stabilized at nearly 100%, indicating that the electrode underwent an effective activation process and achieved highly reversible zinc-ion storage. Notably, after 53 charge–discharge cycles, the specific capacity remained at 315.1 mAh g^−1^, corresponding to a high capacity retention of 92.2%, which reflects excellent long-term cycling stability.

During the first seven cycles, a progressive increase in capacity was observed, which can be attributed to the gradual activation of the electrode material and the associated reduction in charge-transfer resistance. This trend is consistent with the subsequent electrochemical impedance spectroscopy (EIS) results. Compared to MgVO, the Al-MgVO electrode exhibited a slightly delayed activation process. In the first 25 cycles, both materials showed comparable capacities; however, after the 25th cycle, a sharp capacity fading was observed for MgVO, while Al-MgVO maintained a stable capacity with no significant degradation.

This contrast in cycling behavior highlights the critical role of Al^3+^ doping in enhancing structural stability and electrochemical performance. The stable capacity profile of Al-MgVO suggests that the incorporation of Al^3+^ does not excessively occupy Zn^2+^ storage sites, thereby preserving the intrinsic capacity of the host framework. Moreover, the doped Al^3+^ ions are believed to reinforce the lattice structure and suppress structural collapse during repeated Zn^2+^ insertion/extraction processes, ultimately contributing to the outstanding reversibility and cycling durability of the Al-MgVO cathode.

Figure 6b presents the rate capability of MgVO and Al-MgVO at various current densities. At current densities of 0.2, 0.3, 0.5, 1, 3, 5, 10, and 20 A g^−1^, Al-MgVO delivered specific capacities of 313.4, 315.2, 312.3, 307.2, 297, 288.7, 266.2, and 207.9 mAh g^−1^, respectively. When the current density was reduced from 20 A g^−1^ to 10, 5, 3, 1, 0.5, and 0.3 A g^−1^, the capacity recovered to 308.4 mAh g^−1^, indicating excellent structural stability and tolerance to the fast insertion and extraction of Zn^2+^ ions. The outstanding rate capability of Al-MgVO further underscores its superior electrochemical performance in high-rate conditions. In comparison, MgVO also exhibited good rate performance, but Al-MgVO demonstrated enhanced stability and higher specific capacity at higher current densities.

**Figure 7:**
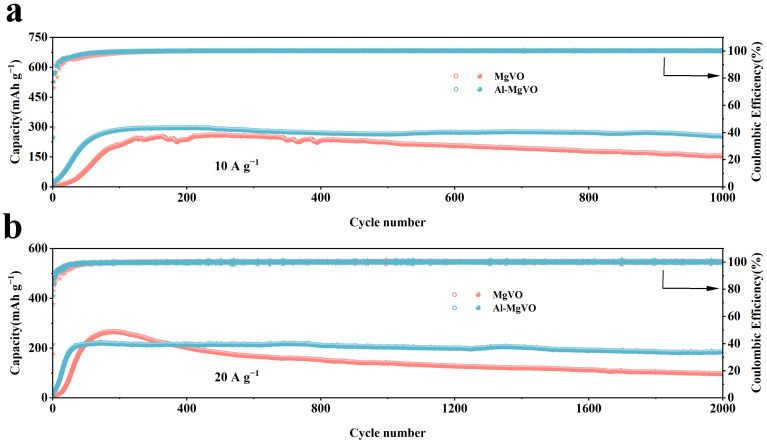
Long-cycle stability of MgVO and Al-MgVO at varying current densities: (**a**) 10 A g^−1^; (**b**) 20 A g^−1^. Open symbols (regardless of shape, such as circles or stars) represent the charge process, while solid symbols indicate the discharge process.

Then the cycling stability of Al-MgVO was systematically evaluated at high current densities of 10 A g^−1^ and 20 A g^−1^, as shown in Figure 7a and Figure 7b, respectively. These evaluations are crucial for assessing the material’s practical applicability in AZIBs. At a current density of 10 A g^−1^, Al-MgVO exhibited an initial discharge capacity of 11.4 mAh g^−1^, and an initial charge capacity of 36 mAh g^−1^. Interestingly, during subsequent cycles, the discharge and charge capacities increased sharply, reaching 271.6 mAh g^−1^, and the Coulombic efficiency (CE) gradually approached 100%. This remarkable capacity enhancement can be attributed to the gradual activation of the electrode material, which is mainly driven by an irreversible phase transition in the electrode. This phase transition led to an increase in the oxidation state of vanadium (V), which subsequently facilitated the higher electrochemical performance of Al-MgVO. After 1000 cycles, the material retained a capacity of 254.3 mAh g^−1^, corresponding to a high capacity retention of 93.6%. This excellent cycling performance demonstrates the material’s potential for high-rate cycling stability and is a clear indication of the favorable influence of aluminum ions in stabilizing the MgVO structure.

Comparatively, the MgVO electrode, after activation, exhibited a relatively higher capacity but suffered from poorer cycling stability, with a faster capacity decay. This suggests that the introduction of Al^3+^ ions into the MgVO structure plays a significant role in enhancing the electrochemical performance and long-term stability of Al-MgVO. The Al^3+^ ions act as a structural pillar, forming stable Al–O bonds with oxygen atoms, thus reinforcing the crystal lattice and preventing structural collapse during cycling. Moreover, aluminum ions do not occupy excessive Zn^2+^ storage sites, which optimizes the electronic structure of MgVO and reduces the interaction between Zn^2+^ and the host material, resulting in more efficient cycling behavior at higher current densities.

At an even higher current density of 20 A g^−1^, Al-MgVO demonstrated an initial discharge capacity of 11.6 mAh g^−1^ and an initial charge capacity of 12.4 mAh g^−1^. After 70 cycles, the discharge and charge capacities significantly improved, reaching 207 mAh g^−1^, with the Coulombic efficiency maintaining a near-perfect value of 100%. After 2000 cycles, the material exhibited a capacity of 186.8 mAh g^−1^, yielding a capacity retention of 90.2%. The sustained high Coulombic efficiency and capacity retention underline the robustness of Al-MgVO under ultra-high current conditions, reinforcing its suitability as a promising cathode material for AZIBs.

The superior cycling stability and high capacity of Al-MgVO at both 10 A g^−1^ and 20 A g^−1^ can be attributed to the structural modifications induced by the incorporation of Al^3+^ ions. These ions not only enhance the structural integrity of the material but also optimize its electrochemical performance by minimizing detrimental side reactions and reducing the impact of Zn^2+^ ion interactions. Furthermore, the ability of Al-MgVO to maintain high Coulombic efficiency and stable capacity over long cycling periods at these elevated current densities is a testament to its potential for high-rate, long-life applications in energy storage technologies.

**Figure 8:**
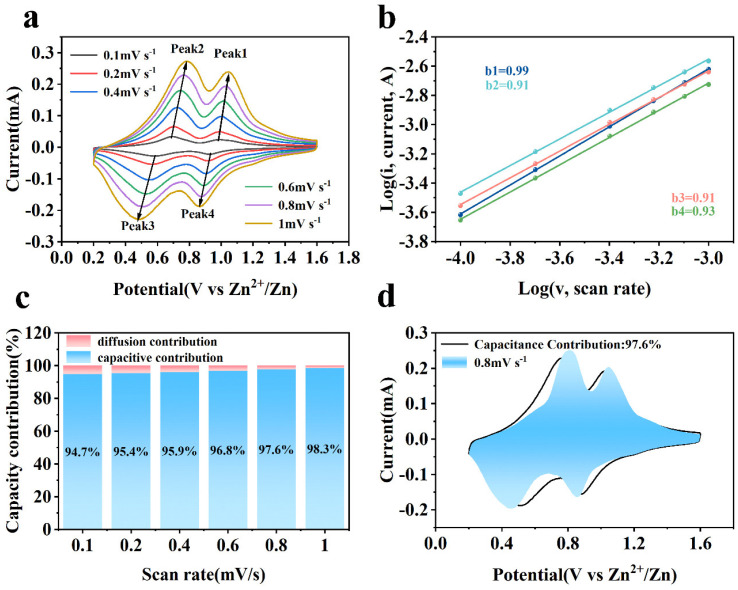
(**a**) CV of Al-MgVO at different sweep speeds; (**b**) *b*-value; (**c**) pseudocapacitance contribution at different scan rates; (**d**) pseudocapacitance contribution at 0.8 mV s^−1^.

To further investigate the outstanding cycling stability and rate performance of Al-MgVO as a cathode material for zinc-ion batteries, CV tests were conducted at various scan rates ranging from 0.1 to 1 mV s^−1^. The CV curves obtained at different scan rates, as shown in Figure 8a, provide valuable insights into the electrochemical behavior of Al-MgVO.

At lower scan rates (0.1 and 0.2 mV s^−1^), the CV curves exhibit well-defined oxidation and reduction peaks, characteristic of the reversible intercalation and de-intercalation of zinc ions. As the scan rate increases, the oxidation peak is observed to shift slightly toward higher potentials, while the reduction peak moves towards lower potentials. This shift can be attributed to the increased influence of kinetic factors at higher scan rates, where the ion diffusion process becomes less efficient. Despite this shift, the overall shape of the CV curves remains well-preserved across all scan rates, which suggests that Al-MgVO maintains its electrochemical reversibility and structural integrity even at higher scan rates. Notably, at the highest scan rate (1 mV s^−1^), the CV curve retains its characteristic shape. This indicates that Al-MgVO is capable of undergoing fast redox reactions while preserving its electrochemical performance. The consistency of the CV profiles at higher scan rates signifies that Al-MgVO can effectively accommodate rapid ion insertion and extraction processes, which is crucial for high-rate performance in energy storage applications.

The observed behavior also implies that Al-MgVO serves as a robust host material that can withstand high discharge rates without significant degradation in performance. The electrochemical stability of Al-MgVO at varying scan rates highlights its potential for fast charge/discharge processes, which is desirable for applications that require both high capacity and high power density. In addition, the results suggest that the insertion of Al^3+^ ions into the MgVO structure enhances the material’s structural stability, allowing it to better endure the stresses induced during rapid cycling. This structural robustness under high scan rates is likely one of the key factors contributing to the superior cycling stability and rate capability of Al-MgVO.

To elucidate the contribution of pseudocapacitance in Al-MgVO, the relationship between peak current (*i*) and scan rate (*v*) was explored through the empirical formula *i* = *av^b^*, where *a* and *b* are adjustable parameters [58]. Typically, the exponent *b* ranges between 0.5 and 1, serving as an indicator of the underlying charge storage mechanism—diffusion-controlled or capacitive-controlled processes [59]. Commonly, a *b* value close to 1 is indicative of capacitive behavior, while a value around 0.5 suggests diffusion control [60,61].

In the studied case of Al-MgVO, the *b* values corresponding to the four redox peaks (Peak1, Peak2, Peak3, Peak4) at various scan rates (0.1, 0.2, 0.4, 0.6, 0.8, and 1 mV s^−1^) were found to be 0.99, 0.91, 0.91, and 0.93, respectively. These values suggest that capacitive behavior predominates in the charge storage process. This is further evidenced by the near-unity *b* values particularly at Peak1, indicating that the redox reactions within the Al-MgVO are primarily governed by the rate of the electrochemical reactions rather than by ionic diffusion.

This dominant capacitive behavior implies that the charge storage mechanism in Al-MgVO is significantly influenced by surface or near-surface electrochemical processes. Such characteristics are beneficial for high-power applications where rapid charge and discharge cycles are crucial. Additionally, the capacitive control of the redox reactions hints at the possibility of engineering the electrode’s surface properties to enhance this behavior, potentially leading to improved cycle stability and rate capability in AZIBs. Moreover, the consistency of the *b* values across different scan rates underscores the robustness of the pseudocapacitive contribution.

To further quantitatively analyze the pseudocapacitive contribution, it is essential to differentiate between capacitive control and diffusion processes, which can be determined based on current contributions. The current can be expressed as follows:

*i* = *k*_1_*v* + *k*_2_*v*^1/2^ (1)

where *i* represents the current, *k*_1_*v* corresponds to the capacitive-controlled contribution, and *k*_2_*v*^1/2^ represents the diffusion-controlled contribution. By calculating the current responses at different voltages and scan rates, the capacitive and ion diffusion contributions can be separated, offering a deeper understanding of the electrochemical behavior.

The pseudocapacitive contribution of Al-MgVO at various scan rates is illustrated in Figure 8c. As the scan rate increases from 0.1 mV/s to 1.0 mV/s, the pseudocapacitive contribution steadily increases, reaching values of 94.7%, 95.4%, 95.9%, 96.8%, 97.6%, and 98.3%, respectively. This significant increase in pseudocapacitive contribution with higher scan rates is indicative of a dominant pseudocapacitive behavior at faster charge/discharge rates. Particularly, at a scan rate of 0.8 mV/s, the pseudocapacitive contribution reaches as high as 97.6% (Figure 8d), signifying that the electrochemical behavior of the Al-MgVO material is primarily governed by pseudocapacitive effects. The dominance of pseudocapacitance suggests high surface reactivity, which is favorable for achieving superior rate capability. High pseudocapacitive contributions are also indicative of an enhanced performance at high current densities and improved cycle stability, as observed in the long-cycle and rate capability tests.

**Figure 9:**
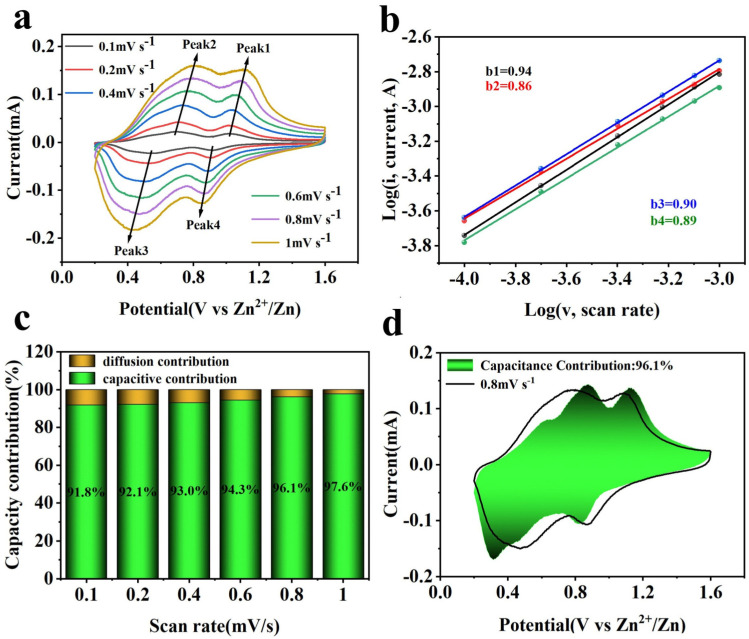
(**a**) CV of MgVO at various scan rates; (**b**) *b*-value analysis; (**c**) pseudocapacitive contribution at different scan rates; (**d**) pseudocapacitive contribution at a scan rate of 0.8 mV s^−1^.

To compare the electrochemical performance of Al-MgVO with MgVO, Figure 9 shows the CV data, *b*-value analysis, and pseudocapacitive contribution for MgVO. When compared to Al-MgVO (Figure 8a), the CV of MgVO exhibits larger potential shifts and significant distortion of the CV curves at higher scan rates. This behavior is indicative of poorer tolerance to high current densities, highlighting the limited electrochemical reversibility and stability of MgVO under fast charge/discharge conditions. The lack of consistency in the CV curve shapes with increasing scan rates suggests that MgVO struggles to maintain structural integrity under rapid cycling.

In contrast, Al-MgVO demonstrates a much more stable CV profile. Despite the higher scan rates, the shape of the CV curves for Al-MgVO remains consistently well-defined, and the redox peaks maintain stable positions throughout, indicating superior structural reversibility. This observation suggests that Al doping enhances the material’s ability to withstand rapid charge/discharge cycles, which is critical for high-rate battery applications. The stability of the oxidation/reduction peaks further confirms the reversible nature of the electrochemical processes in Al-MgVO, showing its ability to maintain high efficiency and performance over extended cycling.

Additionally, the pseudocapacitive contributions for MgVO were analyzed at various scan rates, as shown in Figure 9c. The results demonstrate that as the scan rate increases from 0.1 mV/s to 1.0 mV/s, the pseudocapacitive contribution of MgVO increases steadily from 91.8% to 97.6%. Although this indicates a prominent capacitive contribution, the values are consistently lower than those observed for Al-MgVO, which shows a range of 94.7% to 98.3% at the same scan rates. These higher values in Al-MgVO suggest a stronger capacitive behavior, implying a more significant contribution from surface-controlled processes and faster ion adsorption/desorption kinetics. This enhanced pseudocapacitive contribution in Al-MgVO further indicates its superior electrochemical performance, which can be attributed to the enhanced surface reactivity and improved structural stability due to Al doping.

The reaction kinetics of the Al-MgVO cathode material were investigated using the Galvanostatic Intermittent Titration Technique (GITT). GITT is a technique for evaluating the relationship between diffusion coefficients and charge transfer in electrochemical systems, as described by the following equation:

(2) $$D^{GITT}=\frac{4}{\pi \tau }(\frac{m_{s}V_{M}}{M_{s}S}{)}^{2}(\frac{∆E_{s}}{∆E_{t}}{)}^{2}$$

where *D^GITT^* is the diffusion coefficient, *τ* is the time of the current pulse, *m_s_* is the mass of the electrode, *V_M_* is the molar volume of the active material, *M_s_* is the molar mass of the active material, *S* is the surface area of the electrode, Δ*E_S_* is the steady-state potential change, and Δ*E_t_* is the potential change at the beginning of the current pulse.

**Figure 10:**
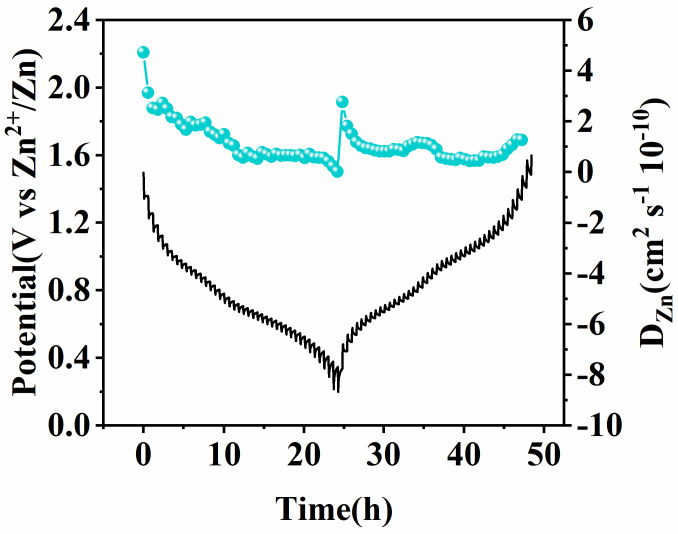
GITT curves and diffusion coefficients of Zn^2+^ for Al-MgVO at 0.1 A g^−1^.

As shown in Figure 10, the diffusion coefficient of Al-MgVO is observed to be in the range of 10^−9^ to 10^−10^ cm^2^ s^−1^. In contrast, the diffusion coefficient of Zn^2+^ in the MgVO material is in the range of 10^−11^ to 10^−12^ cm^2^ s^−1^. This significant difference can be attributed to the Al doping, which increases the electronegativity of the material and enhances its adsorption ability. This, in turn, facilitates a faster charge transfer rate, leading to an accelerated diffusion of Zn^2+^ within the Al-MgVO structure. The increased diffusion rate of Zn^2+^ in Al-MgVO suggests that Al doping not only improves the electrochemical performance by enhancing ion diffusion but also contributes to the material’s superior rate capability. These results provide a clear indication that Al-MgVO exhibits better electrochemical kinetics compared to its MgVO counterpart, which is advantageous for high-performance AZIBs.

To further investigate the influence of Al doping on the conductivity of the MgVO material, EIS was employed to evaluate the charge transfer resistance and ion diffusion resistance of both Al-MgVO and MgVO before and after cycling. The high-frequency region of the EIS spectrum, represented by the semicircular arc, reflects the charge transfer resistance, while the low-frequency region corresponds to the linear section, which represents the ion diffusion resistance.

**Figure 11:**
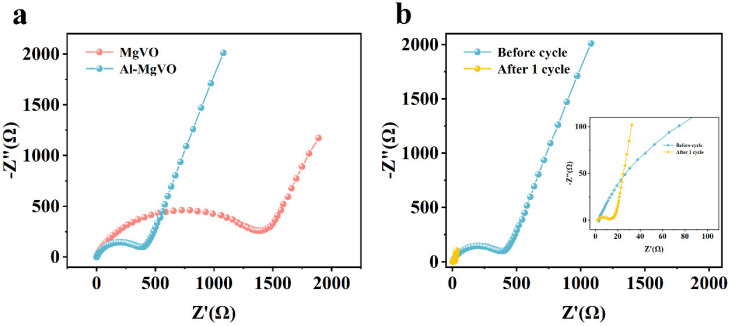
(**a**) Impedance spectra of MgVO and Al-MgVO; (**b**) impedance spectra before and after cycling of Al-MgVO.

The EIS spectra of Al-MgVO and MgVO before cycling are shown in Figure 11a. It can be observed that the charge transfer resistance of Al-MgVO is significantly lower than that of MgVO, suggesting that the insertion of Al^3+^ ions has effectively reduced the resistance of MgVO. Lower charge transfer resistance is favorable for the migration of Zn^2+^ ions, leading to improved rate performance and enhanced cycling stability at high current densities in Al-MgVO. This improvement can be attributed to the incorporation of Al^3+^ ions, which optimize the electronic structure of the material. Furthermore, the formation of stable Al–O bonds between Al and oxygen results in a substantial reduction of the electrostatic interaction between Zn^2+^ ions and the host material, thereby enhancing the conductivity of MgVO and promoting its electrochemical performance.

In addition, the EIS analysis after one cycle was conducted to further investigate the change in charge transfer resistance post-cycling (Figure 11b). The results indicate that the charge transfer resistance of Al-MgVO after one cycle is much lower compared to the pre-cycling resistance. This significant change suggests that the electrochemical activation occurring during the initial cycle plays a crucial role in enhancing the charge transport properties of the Al-MgVO cathode.

This observation is in good agreement with the cycling performance results shown in Figure 6a, where a gradual increase in specific capacity is observed for Al-MgVO during the initial seven cycles. Such behavior can be ascribed to the progressive activation of the electrode material, which facilitates improved ion and electron transport across the electrode–electrolyte interface.

**Figure 12:**
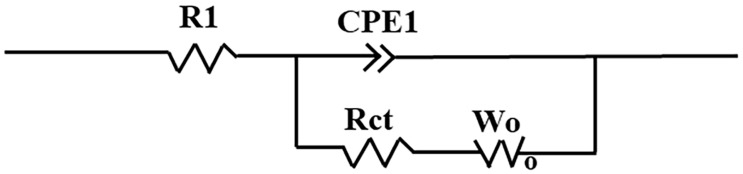
The equivalent electric circuit.

The EIS impedance spectra were fitted according to the equivalent circuit of Figure 12. From the fitted results, the initial charge transfer resistance (*R*_ct_) of Al-MgVO is measured to be 1015.2 Ω, which significantly decreases to 211.9 Ω after 50 charge/discharge cycles. This substantial reduction in *R*_ct_ clearly indicates that the electrode undergoes a notable activation process during cycling. The decreased *R*_ct_ suggests an enhancement in charge transfer kinetics and a reduction in electrode polarization, likely resulting from improved electronic conductivity and better ion diffusion pathways formed through electrochemical activation.

**Figure 13:**
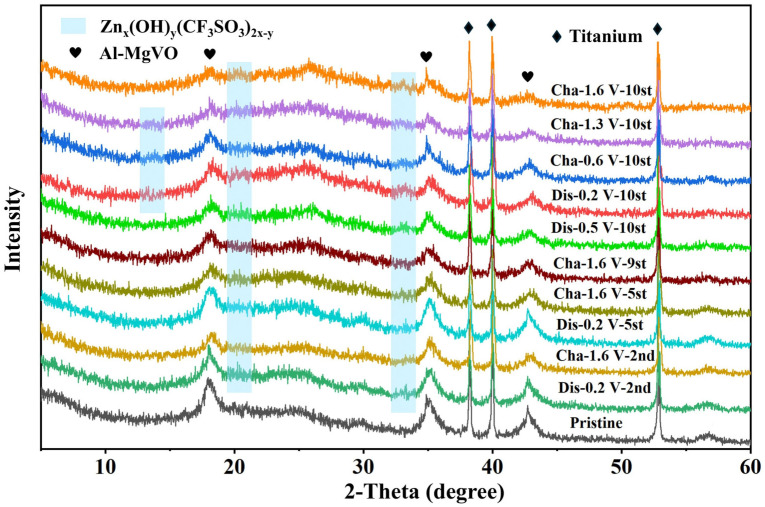
Ex-situ XRD patterns of Al-MgVO after the 2nd, 5th, and 10th full charge/discharge cycles at a current density of 0.1 A g^−1^.

To evaluate the structural evolution of the Al-MgVO electrode during the electrochemical cycling process, ex-situ XRD patterns were recorded after the 2nd, 5th, and 10th charge–discharge cycles at a current density of 0.1 A g^−1^, as shown in Figure 13. The diffraction patterns exhibit negligible changes in the main characteristic peaks of Al-MgVO throughout the cycling process, indicating that the bulk crystal structure remains highly stable. This structural retention demonstrates the excellent structural integrity and robustness of the Al-MgVO framework under repeated Zn^2+^ insertion/extraction, which is crucial for long-term cycling stability.

Interestingly, several additional weak diffraction peaks emerge at 2θ values of approximately 13.3°, 20.4°, and 33.2° during the 2nd, 5th, and 10th cycles. These peaks are consistent with the formation of Zn_x_(OH)_y_(CF_3_SO_3_)_2x−y_, a common byproduct attributed to proton (H^+^) insertion during the discharge process, as reported in the previous literature [62]. The presence of these peaks suggests that minor side reactions involving electrolyte decomposition or competitive H^+^ intercalation may occur during the Zn^2+^ storage process.

However, these impurity-related peaks disappear when the electrode is fully charged to 1.6 V, implying that the formation of Zn_x_(OH)_y_(CF_3_SO_3_)_2x−y_ is reversible and does not lead to permanent structural degradation. This reversible behavior highlights the good electrochemical reversibility of the Al-MgVO electrode and confirms that the observed side reactions are dynamically reversible and do not compromise the structural stability or the long-term performance of the material.

As summarized in Table 2, the electrochemical performance of Al-MgVO cathodes compares favorably with that of several representative cathode materials reported for AZIBs, including vanadium-based oxides (e.g., hydrated V_2_O_5_), manganese oxides (e.g., Mn_2_O_3_ and ZnMn_2_O_4_), and Prussian blue analogs (e.g., ZnHCF). For instance, although V_2_O_5_·nH_2_O delivers a high initial capacity, its layered structure lacks mechanical robustness, leading to rapid capacity fading and structural degradation under high current densities. Mn_2_O_3_-based materials exhibit moderate rate performance but suffer from limited cycling stability due to Mn dissolution and framework collapse [63]. Spinel-type ZnMn_2_O_4_ offers improved structural stability; however, its capacity retention under ultrafast cycling conditions remains moderate [64]. Likewise, ZnHCF benefits from an open framework conducive to Zn^2+^ diffusion but is hindered by relatively low capacity (<100 mAh g^−1^) and poor stability in mildly acidic electrolytes [65].

In comparison, the Al-MgVO cathode exhibits a well-balanced electrochemical performance, characterized by both high specific capacity and outstanding long-term cycling stability. Remarkably, it maintains 90.2% of its initial capacity after 2000 cycles at a high current density of 20 A g^−1^, surpassing the performance of most benchmark cathode materials reported to date. This enhanced performance is primarily attributed to the synergistic effects of Al^3+^ doping, which not only stabilizes the spinel framework but also reduces the electrostatic interaction between Zn^2+^ ions and the host lattice. Consequently, both structural robustness and Zn^2+^ ion transport kinetics are significantly improved. These results highlight the potential of Al-MgVO as a durable and scalable cathode material for high-rate AZIBs.

## 3. Materials and Methods

### 3.1. Preparation of Material

The spinel MgVO was synthesized using the sol-gel method as follows: 2 mmol of magnesium acetate tetrahydrate and 4 mmol of vanadium acetylacetonate were dissolved in 200 mL of anhydrous ethanol and subjected to ultrasonic treatment for 30 min. The resulting solution was then stirred at room temperature at 600 rpm for 3 h. Subsequently, the mixture was placed in a water bath, and the solvent was evaporated at 80 °C to yield the MgVO precursor. The obtained solid was dried in an electric drying oven at 60 °C for 12 h, followed by grinding into a fine powder. Finally, the powder was calcined in an argon atmosphere at 800 °C for 12 h with a heating rate of 2 °C/min to obtain the MgVO product.

To synthesize Al-MgVO, 0.5 mmol of aluminum nitrate nonahydrate, 2 mmol of magnesium acetate tetrahydrate, and 4 mmol of vanadium acetylacetonate were dissolved in 200 mL of anhydrous ethanol. The mixture was then subjected to a synthesis procedure similar to that used for the preparation of MgVO, resulting in the formation of Al-MgVO.

In our preliminary experiments, we explored various Al doping concentrations to optimize the electrochemical performance of the MgVO-based cathode. We found that at low Al doping levels, the substitution of Mg^2+^ by Al^3+^ was minimal, leading to electrochemical behavior similar to undoped MgVO. This was attributed to the insufficient reduction in the electrostatic interaction between Zn^2+^ and the host lattice, which limited ion diffusion and charge transfer kinetics. On the other hand, at high Al doping levels, the excessive incorporation of Al^3+^ significantly disrupted the MgVO spinel structure, resulting in inferior electrochemical performance due to the formation of a different structural phase.

The Al doping level used in this study represents the optimal concentration identified through our trials, where Al^3+^ partially replaces Mg^2+^ ions without compromising the spinel framework. This substitution leads to the formation of Al–O bonds, which stabilize the crystal structure and reduce the electrostatic interaction between Zn^2+^ and the host lattice, ultimately enhancing the material’s electrochemical performance.

### 3.2. Materials Characterization

The XRD measurements were conducted using Cu Kα radiation with a Smart Lab SE system (Tokyo, Japan), providing detailed insights into the crystalline structure of the materials. Morphological analyses were performed using scanning electron microscopy (SEM) to examine the surface features of the materials. SEM images were obtained with a Hitachi SU8010 microscope (Tokyo, Japan), enabling high-resolution visualization of the material surfaces.

The elemental distribution within the synthesized materials was evaluated using EDS integrated into the SEM system. This technique facilitated qualitative analysis of the elemental composition at various points across the samples, offering insights into the uniformity and purity of the materials. Additionally, the elemental composition was determined quantitatively using an ICP optical emission spectrometer (ICP-OES, Optima 8000, Waltham, MA, USA).

The XPS was further employed to investigate the elemental composition and to monitor changes in the oxidation states of the constituent elements. These analyses were carried out using a Thermo ESCALAB 250Xi spectrometer (Waltham, MA, USA), which provided high-resolution spectral data for both powder samples and sliced electrode materials. This technique was particularly useful for understanding the electronic environment of the elements and tracking changes associated with electrochemical processes.

### 3.3. Electrode Fabrication

The cathode material (MgVO), conductive agent (acetylene black), and binder (polyvinylidene fluoride, PVDF) were mixed in a mass ratio of 6:3:1 and placed into a mortar for uniform blending. The mixture was ground continuously until a homogeneous consistency was achieved, at which point N-methylpyrrolidone (NMP) was incrementally added to form a uniform slurry. The slurry was kneaded continuously until a glossy appearance was observed. Subsequently, the slurry was coated onto titanium foil (thickness: 0.03 mm) and vacuum-dried at 110 °C for 12 h. After drying, the titanium foil was punched into electrode discs with a diameter of 10 mm, achieving an active material loading of approximately 1–1.6 mg cm^−2^.

For the coin cell assembly, zinc foil (0.1 mm) was employed as the anode, and 3M Zn(CF_3_SO_3_)_2_ solution was used as the electrolyte. A glass fiber separator was incorporated, with titanium foil serving as the current collector. The synthesized cathode material was utilized as the positive electrode. The coin cell was assembled under ambient air conditions at room temperature, followed by sealing using a sealing machine.

### 3.4. Electrochemical Measurements

The CV analyses were performed using a coin cell configuration on a CHI 760E electrochemical workstation. The cathode sheet, coated with the active material, served as the working electrode and was evaluated within a voltage range of 0.2 V to 1.6 V (vs. Zn/Zn^2+^) at scan rates of 0.1, 0.2, 0.4, 0.6, 0.8, and 1 mV s^−1^. The EIS measurements were conducted by applying a small-amplitude sinusoidal AC signal, enabling the assessment of the system’s impedance. The acquired data were analyzed using equivalent circuit modeling on the CHI 760E, with a frequency range from 0.01 Hz to 100 kHz and a voltage amplitude of 5 mV. The GITT was utilized to investigate the diffusion processes and the interaction between charge transfer and electrochemical reactions at the electrode surface. This method involved cycles of pulse application, constant current, and relaxation, which allowed for the determination of the chemical diffusion coefficient. These tests were conducted using the CT2001A Battery Test System (Wuhan LAND Electric Co., Wuhan, China). Furthermore, electrochemical cycling and rate capability tests were carried out at room temperature on coin cells at varying current densities ranging from 0.1 to 20.0 A g^−1^, employing the same CT2001A system. Collectively, these methodologies facilitated a comprehensive evaluation of the electrochemical properties of the examined electrodes.

## 4. Conclusions

In conclusion, this study highlights the successful development of Al-doped MgVO spinel cathodes for AZIBs, addressing key challenges such as poor ion diffusion and structural instability. By pre-inserting Al^3+^ ions into the spinel structure via a sol-gel method, the Al-doped MgVO cathode demonstrates significant improvements in both structural stability and electronic conductivity. The Al^3+^ ions form strong Al–O bonds, which reduce the electrostatic interactions between Zn^2+^ ions and the host material, thereby facilitating more efficient Zn^2+^ intercalation and deintercalation.

The Al-MgVO cathode exhibits excellent electrochemical performance, particularly at high current densities. At 10 A g^−1^, the material maintains a capacity of 254.3 mAh g^−1^ after 1000 cycles, with a remarkable capacity retention of 93.6%. More importantly, at an ultra-high current density of 20 A g^−1^, it retains a capacity of 186.8 mAh g^−1^ after 2000 cycles, with a capacity retention of 90.2%, making it an outstanding candidate for high-rate applications in large-scale energy storage systems. These results suggest that Al-doped MgVO cathodes offer a promising solution for enhancing the performance of AZIBs, demonstrating the potential for sustainable, high-rate energy storage. Further optimization of this material could lead to significant improvements in the performance and viability of AZIBs for large-scale applications.

## Figures and Tables

**Table 1:** ICP measurements results: molar ratios of elements in Al-MgVO and MgVO.

Sample	Magnesium (mg/kg) × 10^4^	Vanadium (mg/kg) × 10^4^	Aluminum (mg/kg) × 10^4^	
Al-MgVO	10.98	37.24	2.58	Mg/V/Al: 1.23/2/0.26
MgVO	9.91	39.24	–	Mg/V:1.02/2

**Table 2:** Comparison of the electrochemical performances over previous reported cathode materials in AZIBs.

Cathode Meterials	Electrochemical Performance (Capacity Retention, Cycle Numbers)	Reference
Al-MgVO	186.8 mAh g^−1^ at 20 A g^−1^ (90.2%, 2000 cycles)	This work
V_2_O_5_	224 mAh g^−1^ at 0.1 mA g^−1^ (75%, 400 cycles)	[41]
Mn_2_O_3_	246 mAh g^−1^ at 0.05 A g^−1^ (88%, 600 cycles)	[63]
ZnMn_2_O_4_	131 mAh g^−1^ at 1 A g^−1^ (85%, 400 cycles)	[64]
ZnHCF	59.2 mAh g^−1^ at 0.3 A g^−1^ (80%, 200 cycles)	[65]
MgV_2_O_6_·1.7H_2_O	259.4 mAh g^−1^ at 4 A g^−1^ (100%, 1500 cycles)	[66]
Na_0.33_V_2_O_5_	218.4 mAh g^−1^ at 1 A g^−1^ (100%, 1000 cycles)	[67]
MnV_2_O_4_@C	240 mAh g^−1^ at 5 A g^−1^ (82%, 2000 cycles)	[68]
Ca_0.67_V_8_O_20_·3.5H_2_O	214 mAh g^−1^ at 5 A g^−1^ (74%, 2000 cycles)	[69]
Ni_0.25_V_2_O_5_·nH_2_O	218 mAh g^−1^ at 5 A g^−1^ (98%, 1200 cycles)	[70]
Zn_0.34_V_2_O_5_.0.37H_2_O	133 mAh g^−1^ at 8 A g^−1^ (85.2%, 1000 cycles)	[71]
Cu_0.95_V_2_O_5_	200 mAh g^−1^ at 5 A g^−1^ (92%, 1000 cycles)	[72]
K_2_V_3_O_8_@GO	161.2 mAh g^−1^ at 5 A g^−1^ (71.7%, 2000 cycles)	[73]

## Data Availability

The data presented in this study are available on request from the corresponding author.
